# Synthesis, Characterization, Molecular Docking, and Preliminary Biological Evaluation of 2-((4-Morpholino-1,2,5-thiadiazol-3-yl)oxy)benzaldehyde

**DOI:** 10.3390/molecules31030574

**Published:** 2026-02-06

**Authors:** Mokete Motente, Uche A. K. Chude-Okonkwo

**Affiliations:** 1 Institute for Artificial Intelligent Systems, University of Johannesburg, P.O. Box 524, Johannesburg 2092, South Africa; 2 Department of Chemical Sciences, University of Johannesburg, P.O. Box 524, Johannesburg 2092, South Africa

**Keywords:** artificial intelligence, 3-chloro-4-morpholino-1,2,5-thiadiazole, 2-hydroxybenzaldehyde, biological evaluation

## Abstract

This study details the synthesis, characterization, molecular docking and preliminary biological evaluation of a new heterocyclic compound, 2-((4-morpholino-1,2,5-thiadiazol-3-yl)oxy)benzaldehyde. This molecule was designed using an artificial intelligence (AI)-based molecular generative model. It was synthesized through a nucleophilic substitution between 3-chloro-4-morpholino-1,2,5-thiadiazole and 2-hydroxybenzaldehyde. Structural elucidation was performed using ^1^H NMR, ^13^C NMR, Elemental Analysis, and Single Crystal X-ray diffraction. AI-guided in silico predictions suggested promising pharmacophoric features and potential biological activity. Preliminary biological evaluation, primarily through anticancer assays, demonstrated moderate to significant activity, supporting further investigation. The findings therefore suggest that this AI-generated molecule could serve as a lead scaffold for developing drugs targeting cancer and other infectious diseases.

## 1. Introduction

The drug development process has a long history that spans millions of years, starting with natural products and evolving into a complex, multi-step process involving various scientific fields. However, the first significant application of drug discovery can be traced back to the 19th century, a time when most nations began relying on medicinal chemists to develop potential drugs [1]. Drug discovery and development is an expensive and time-consuming process, emphasizing the need for advanced tools. To overcome this challenge, scientists have turned to tools like machine learning and artificial intelligence to improve the drug development process [2].

However, one challenge of using AI to discover new molecules is the difficulty in synthesizing many AI-generated molecules in the laboratory [3,4]. Therefore, while the AI-based approach to drug discovery provides a strong method for exploring the vast chemical space for potential drug molecules, the synthesizability of the resulting molecules is essential.

Among various diseases, cancer treatment stands to benefit significantly from AI-driven drug discovery. Cancer is one of the leading causes of death worldwide, and so far, chemotherapy remains the primary cancer treatment globally [5,6], although it has some limitations; hence, there is a need to develop new cancer therapies. This paper reports the synthesis, characterization, and preliminary bioactivity evaluation of 2-((4-morpholino-1,2,5-thiadiazol-3-yl)oxy)benzaldehyde, an AI-generated molecule designed using a fragmentation-and-build computational approach in which known bioactive molecules are decomposed into smaller building blocks, recombined to generate new structures, and evaluated in silico for drug-likeness and biological relevance prior to experimental synthesis and validation.

The molecule, 2-((4-morpholino-1,2,5-thiadiazol-3-yl)oxy)benzaldehyde, comprises the morpholine ring, 1,2,5-thiadiazole ring, and the benzaldehyde moiety. The morpholine ring (tetrahydro-1,4-oxazine) is a heterocyclic molecule containing an oxygen and nitrogen atom and possesses proven physicochemical, biological, and metabolic properties [7,8]. Preludin is the first morpholine-containing drug to be used for medicinal purposes, and it was used for the treatment of obesity [9], and a couple of years later, more morpholine drugs were developed, and utilized widely as analgesics, anti-inflammatory, anticancer, antidepressants, HIV-protease inhibitors, antimicrobial and anti-viral agents [10,11]. Since then, significant progress has been made towards exploring the full spectrum of the biological functionality of morpholine-containing compounds, and this study is a continuation of such exploration.

The 1,2,5-thiadiazole moiety is a planar, heterocyclic, aromatic five-membered compound containing two nitrogen atoms and a sulfur atom with a lone pair. The 1,2,5-thiadiazole derivatives have been utilized in various applications, particularly in drug development, and have demonstrated remarkable effectiveness across different therapeutic areas, including antiprotozoal, antidiabetic, antioxidant, and anticancer activities [12,13]. Molecules containing 1,2,5-thiadiazole derivatives have also exhibited anti-diabetic activity on key enzymes like α-glucosidase and α-amylase, providing new therapeutic options for diabetes management [14,15]. Additionally, they have demonstrated antioxidant potential, and reports indicate their role in fighting oxidative-stress-related diseases [16,17]. Furthermore, the anticancer activity of these derivatives has yielded promising results against various cancer cell lines, as shown in multiple studies [18,19]. Therefore, the synthesis of thiadiazole derivatives, characterized by diverse methods and significant pharmacological potential, remains a prominent focus in drug discovery and development.

The benzaldehyde moiety in the molecule is Salicylaldehyde (2-hydroxybenzaldehyde), the simplest hydroxybenzaldehyde derivative with a hydroxyl group at the ortho position to the aldehyde group. It has significant potential in drug discovery due to its diverse biological activities and versatility in synthetic chemistry, serving as a key scaffold in designing bioactive compounds, especially in anti-inflammatory, antioxidant, antimicrobial, and anticancer therapies [20,21]. Salicylaldehyde and, more notably, its derivatives are of interest in anticancer drug development due to their chemical versatility and reported cytotoxic activity. Its structural modification, particularly through conjugation with heterocycles, has led to compounds that inhibit cancer cell growth via mechanisms such as apoptosis induction and oxidative stress modulation, making salicylaldehyde a valuable scaffold for anticancer drug design [22,23]. In addition, the reported anticancer potential of heterocycle-conjugated salicylaldehyde derivatives motivated their incorporation into the design of the ligand investigated in this study.

Considering the above discussion about the different structural motifs of the AI-generated molecules, it can be inferred that the molecule may have potential applications as an anticancer, antimicrobial, or enzyme inhibition agent. Hence, we performed molecular docking analysis and bioactivity studies of the 2-((4-morpholino-1,2,5-thiadiazol-3-yl)oxy)benzaldehyde against breast and prostate cancer cell cultures, respectively.

## 2. Results and Discussion

### 2.1. Chemistry

The parent molecule, namely 2-((4-morpholino-1,2,5-thiadiazol-3-yl)oxy)benzaldehyde, was successfully synthesized by reacting 3-chloro-4-morpholino-1,2,5-thiadiazole and Salicylaldehyde in anhydrous DMF (solvent) in the presence of potassium carbonate (K_2_CO_3_) as a base. The base facilitates the deprotonation of the Salicylaldehyde moiety, resulting in the formation of an ether bond (C-O-C) between the two moieties upon their reaction. The chemical structure of the synthesized molecule was validated and confirmed by standard Spectrometric techniques such as ^1^H NMR and ^13^C NMR, and further characterized by elemental analysis (EA) and Single Crystal X-ray Diffraction (SC-XRD). ^1^H NMR and ^13^C NMR spectroscopy provided information on the hydrogen and carbon environments, confirming the molecular framework and functional group connectivity.

Additionally, elemental analysis (CHNS) verified the compound’s empirical composition by comparing the measured carbon, hydrogen, nitrogen, and sulphur content with theoretical values, thereby supporting the proposed molecular formula. Finally, single-crystal X-ray diffraction (SC-XRD) gave direct insight into the three-dimensional arrangement of atoms, unambiguously confirming the molecular structure.

### 2.2. Nuclear Magnetic Resonance Spectroscopy

**Figure 1:**
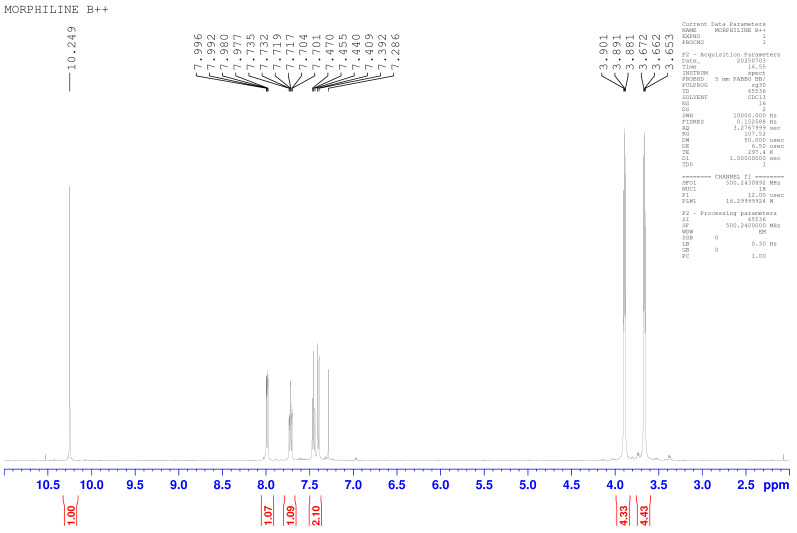
^1^H NMR spectra for the parent molecule: 2-((4-morpholino-1,2,5-thiadiazol-3-yl)oxy)benzaldehyde.

As mentioned earlier, the parent molecule was characterized by proton NMR to confirm the molecular structure. The ^1^H NMR spectra displayed a singlet at δ 10.24 ppm that corresponded to the aldehyde proton (–CHO) of the benzaldehyde moiety. The aromatic protons of the substituted benzene ring appeared as a multiplet between δ 7.996–7.286 ppm, integrating for four protons. The morpholine ring showed two sets of triplets at δ 3.901 ppm and δ 3.653 ppm, representing the O–CH_2_ and N–CH_2_ methylene protons, respectively. Thus, the integration and multiplicity of the observed signals aligned with the proposed structure, and Figure 1 shows the ^1^H NMR spectra for the parent molecule.

The molecule was further characterized by ^13^C NMR, and the spectra showed distinct resonances corresponding to the aromatic, heterocyclic, morpholine, and aldehydic carbons. Signals in the range δ 150–155 ppm are attributed to the C=N and C–O carbons of the thiadiazole–oxygen linkage. The aldehyde carbon appears as a characteristic downfield signal at approximately δ 185–190 ppm. Aromatic ring carbons resonate between δ 122–135 ppm, consistent with substituted benzaldehyde derivatives.

**Figure 2:**
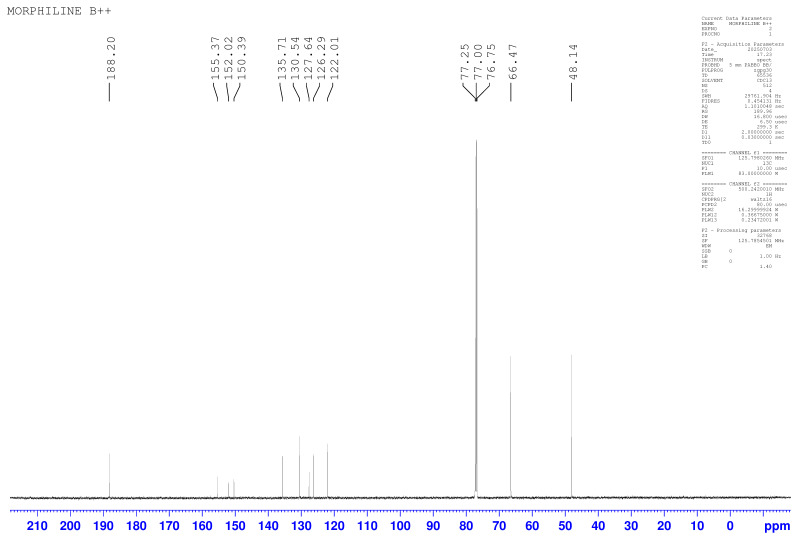
^13^C NMR spectra for the parent molecule 2-((4-morpholino-1, 2, 5-thiadiazol-3-yl) oxy) benzaldehyde.

The morpholine carbons give signals in the δ 60–80 ppm region, corresponding to the N–CH_2_ and O–CH_2_ environments. The chemical shift distribution confirms the proposed molecular structure and the successful formation of the thiadiazole–aryl ether linkage. Shown in Figure 2 is the ^13^C NMR spectrum for the parent molecule: 2-((4-morpholino-1,2,5-thiadiazol-3-yl)oxy)benzaldehyde.

### 2.3. Single Crystal XRD (SC-XRD) Analysis

As mentioned earlier, the molecule was successfully characterized by SC-XRD, and the structure is reported in a crystallography-based manuscript [24]. The successful structural determination of the molecule provides valuable insights into the spatial arrangement of atoms and further confirms the coordination mode of the molecule, thereby supporting the characterization data obtained.

### 2.4. Biological Study

The synthesized compound, 2-((4-morpholino-1,2,5-thiadiazol-3-yl)oxy)benzaldehyde, was evaluated for its cytotoxic activity against prostate (PC-3) and breast (MCF-7) cancer cell lines. Dichloromethane (DCM), the solvent used to dissolve the compound, was included as a negative control, while camptothecin served as a positive control. The compound exhibited IC_50_ values of 13.11 μM and 16.34 μM against PC-3 and MCF-7 cells, respectively. In contrast, no significant cytotoxic effect was observed in non-cancerous MRC-5 fibroblasts at the same concentration, suggesting preliminary differential antiproliferative activity and supporting its potential for further investigation. According to the National Cancer Institute (NCI) criteria, compounds exhibiting IC_50_ values ≤ 30 μM are classified as highly potent anticancer agents [25], therefore the observed cytotoxic response warrants further investigation.

Morpholine-containing scaffolds are recognized for their role in enhancing drug solubility, cellular permeability, and receptor binding affinity, which collectively contribute to improved pharmacological profiles [26,27]. Similarly, 1,2,5-thiadiazole-containing compounds have been reported to exhibit cytotoxic, antimicrobial, and antioxidant activities; however, these effects are highly dependent on the specific molecular structure, and their underlying mechanisms remain unclear [28].

Comparative studies by Alarcón-Espósito et al. (2021) on morpholine-linked heterocycles reported IC_50_ values in the range of 15–25 μM against MCF-7 and PC-3 cells, which aligns closely with the potency observed in this study [29]. Moreover, thiadiazole analogues bearing benzaldehyde or ether linkages have been shown to exhibit enhanced cytotoxic activity (IC_50_ = 12–20 μM), primarily due to improved interaction with nucleophilic residues in target proteins [30]. Therefore, the synergistic combination of the morpholine and 1,2,5-thiadiazole moieties within a benzaldehyde framework in the current molecule likely underpins its potent and selective anticancer activity.

Camptothecin was used as a positive control due to its well-established anticancer activity. Under the conditions tested, the compound showed greater inhibitory effects than Camptothecin in both prostate and breast cancer cell lines. While this observation is encouraging, the comparison is limited by experimental conditions and differences in mechanisms of action, and the results should therefore be regarded as preliminary.

**Figure 3:**
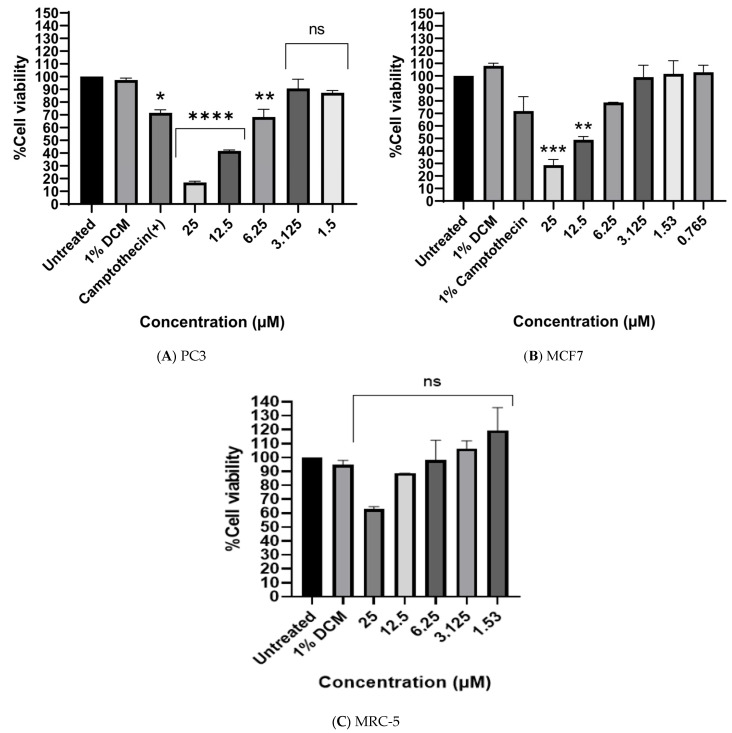
Effect of the parent compound on (**A**) PC-3, (**B**) MCF-7, and (**C**) non-cancerous MRC-5 fibroblast cells. Dichloromethane (1% DCM) was used as the solvent/negative control, and camptothecin served as the positive control. Statistical analysis was performed using GraphPad Prism 8.4.2 with one-way ANOVA, and error bars represent mean ± SD from three independent experiments. The asterisks (*) signify the degree of significance, whereby (**) means moderately significant, (***) means highly significant, and (****) means very highly significant, while ns indicates no significant difference.

Overall, these findings suggest that 2-((4-morpholino-1,2,5-thiadiazol-3-yl)oxy)benzaldehyde exhibits cytotoxic activity within the expected range [25]. Although definitive selectivity has not been established, the observed differential response between cancerous and non-cancerous cells supports further investigation of this scaffold, including structural optimization and mechanistic studies targeting apoptosis, oxidative stress modulation, or receptor-specific interactions in prostate and breast cancer models. Figure 3 shows the effect of the compound on (A) PC-3, (B) MCF-7, and (C) non-cancerous MRC-5 fibroblast cells, with 1% DCM serving as the solvent/negative control and camptothecin as the positive control.

### 2.5. Molecular Docking Studies

**Figure 4:**
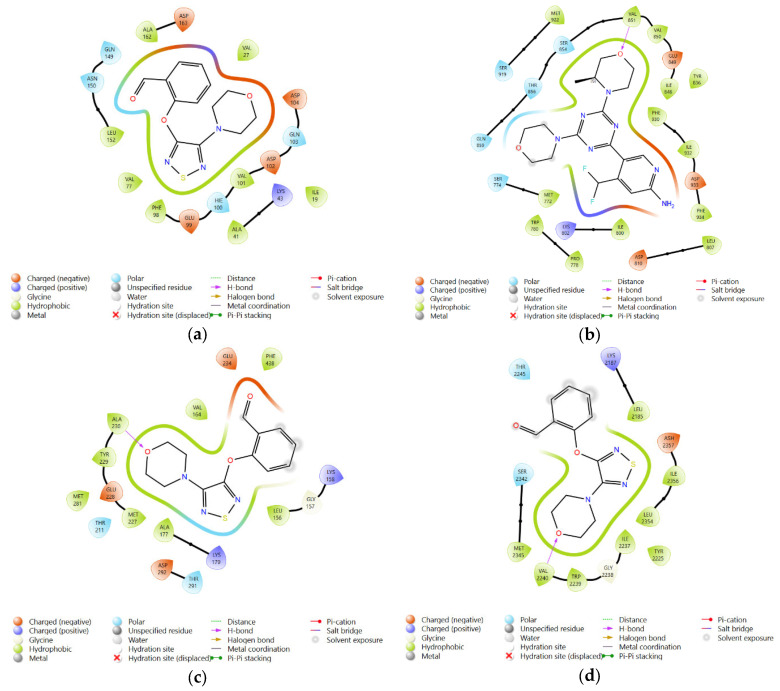
Ligand-protein interactions in stick representation for (**a**) 5L2S, (**b**) 6OAC, (**c**) 4GV1, and (**d**) 4JSV, generated using Schrödinger Suite Release 2025-3 (Schrödinger, LLC, New York, NY, USA). Hydrogen bonds are shown as dashed lines, and hydrophobic contacts are colored surfaces. Metal ions and some residues share the same color because the software assigns colors automatically, and this does not indicate any chemical or interaction property.

The molecular docking scores for 2-((4-morpholino-1,2,5-thiadiazol-3-yl)oxy)benzaldehyde against the protein targets 5L2S, 6OAC, 4GV1, and 4JSV were −6.02, −7.68, −4.72, and −6.34 kcal/mol, respectively. The corresponding ligand–protein interaction diagrams are presented in Figure 4. These docking results suggest moderate binding interactions with the selected cancer-related targets. Analysis of the interaction patterns provides insights that may be useful for future structural optimization of the ligand to improve binding affinity and further explore its anticancer potential.

## 3. Materials and Methods

### 3.1. Chemicals and Reagents

All chemical reagents used during this study were of reagent grade and were utilized without any further purification, while solvents were of analytical grade, and they were also utilized without any further purification.

### 3.2. Instrumentation

The ^1^H and ^13^C NMR spectra were recorded using a Bruker spectrometer (Billerica, MA, USA) operating at 500 and 125 MHz, respectively. The NMR data were collected at room temperature, and the chemical shifts were reported in parts per million (ppm) units, with tetramethylsilane (TMS) used as the internal standard.

### 3.3. AI-Enabled Molecular Generation

**Figure 5:**
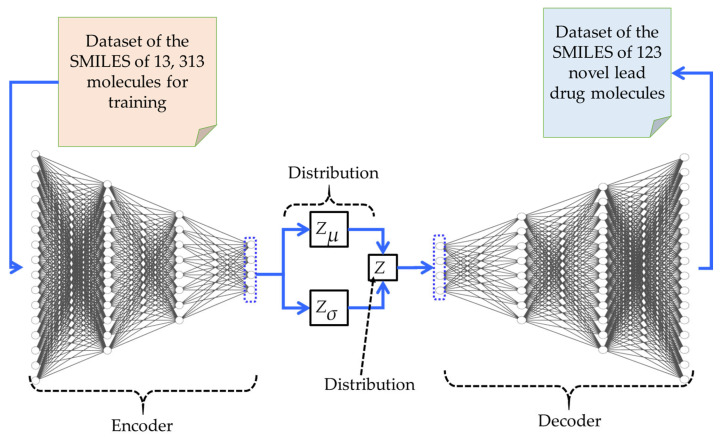
An in silico AI-based framework for generating the molecule under study.

**Figure 6:**
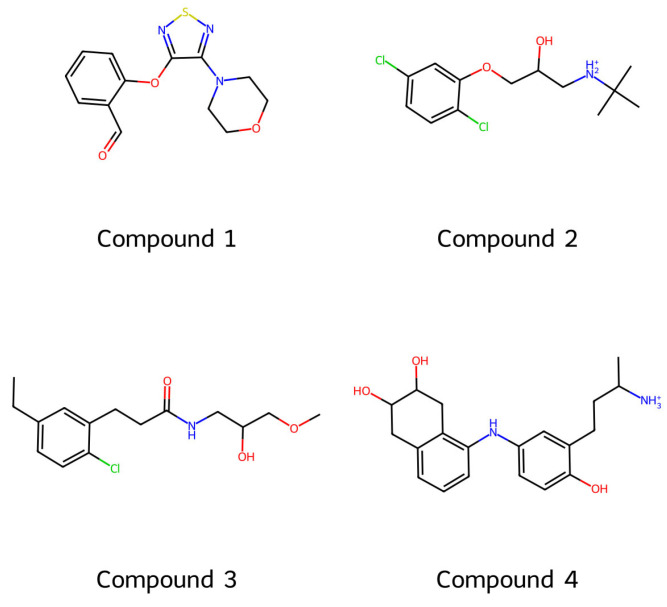
Sample of the AI-generated compounds (Compound **1** is 2-((4-morpholino-1,2,5-thiadiazol-3-yl)oxy)benzaldehyde).

The 2-((4-morpholino-1,2,5-thiadiazol-3-yl)oxy)benzaldehyde is a part of a set of 123 virtual leads generated using a generative model. Figure 5 illustrates the AI-based framework used to generate the molecules. Within this framework, a set of 13,313 molecules synthesized using the fragmentation-and-build computational method was utilized to train a variational autoencoder (VAE)-based generative model. The VAE structure consists of an encoder, a decoder, and a latent space defined by the mean, *Zμ*, and standard deviation, *Zσ*, of a Gaussian distribution. The output of this process is a set of 123 unique molecules generated by the VAE-based generative model. All 123 molecules passed the drug-likeness and synthesizability tests, which were conducted using the RDKit Python (open source cheminformatics software, version 2023.09.5) library. A sample of the AI-generated compounds, including Compound **1** (2-((4-morpholino-1,2,5-thiadiazol-3-yl)oxy)benzaldehyde), is shown in Figure 6, while Figure 5 portrays an in silico AI-based framework for generating the molecule under study.

### 3.4. Synthesis of 2-((4-Morpholino-1,2,5-thiadiazol-3-yl)oxy)benzaldehyde

**Scheme 1:**
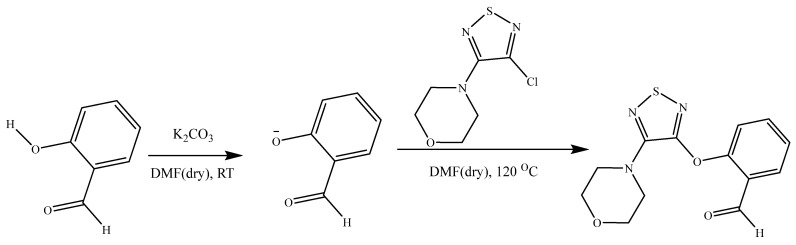
Reaction pathway for the synthesis of the parent molecule.

To synthesize the title compound, a mixture of salicylaldehyde (200 mg, 1.64 mmol) and potassium carbonate (276 mg, 2.0 mmol) was dissolved in 8 mL of anhydrous N, N-dimethylformamide (DMF) under a nitrogen atmosphere and stirred at room temperature for 30 min to generate the corresponding phenoxide ion. To this stirred solution, 3-chloro-4-morpholino-1, 2, 5-thiadiazole (1.82 mmol) was added in one portion, and the reaction mixture was then heated at 120 °C for 18 h. The progress of the reaction was monitored by thin-layer chromatography (TLC) using a hexane: ethyl acetate (8:2) solvent system. Upon completion, the reaction mixture was cooled to room temperature and poured into 50 mL of ice-cold water, followed by extraction with ethyl acetate (3 × 20 mL). The combined organic layers were washed with brine, dried over anhydrous sodium sulphate, and concentrated under reduced pressure. The resulting crude product was purified by silica gel column chromatography using a gradient of hexane and ethyl acetate as eluent, affording 2-((4-morpholino-1, 2, 5-thiadiazol-3-yl) oxy) benzaldehyde as a pale yellow solid [31]. The yellow solid was re-crystallised by slow evaporation of acetone, resulting in the formation of pale yellow crystals suitable for X-ray analysis. Scheme 1 below shows the reaction pathway for the synthesis of the parent molecule. Yield: 58%, Elemental analysis: (Chemical formulae: C_13_H_13_N_3_O_2_S; Molecular weight: 291.33; Calculated expected outcome: C, 53.60%; H, 4.50%; N, 14.42%; O, 16.48%; S, 11.00%. Found in elemental analysis: C, 55.3%; H, 4.5%; N, 16.0%; S, 11.9%. ^1^H NMR (500 MHz, chloroform-*d*_1_): δ 10.25 (s, 1H), 7.99 (dd, *J* = 7.7, 1.7 Hz, 1H), 7.80–7.61 (m, 1H), 7.57–7.36 (m, 2H), 4.02–3.80 (t, 4H), 3.80–3.54 (t, 4H). ^13^C NMR (500 MHz, chloroform-*d*_1_): δ 188.20, 155.37, 152.04, 150.39, 135.71, 126.29, 122.01, 77.25, 77.00, 76.75, 66.47, 49.14.

### 3.5. Single Crystal X-Ray Diffraction Analysis

A Bruker Apex II 4K diffractometer (Bruker AXS Inc., Madison, WI, USA) was used for crystal data collection using graphite monochromated Mo Kα radiation (λ = 0.709626 Å) with ω-and φ-φ-scans at 100(2) K. All reflections were merged and integrated with SAINT-PLUS, and SADABS [32] was used to correct Lorentz, polarization, and absorption effects. The heavy atom method was used to solve the structures and refined through full-matrix least squares cycles using Shelx-97 [33] as part of the WinGX [34] package, with ∑(║Fo│ − │Fc║)^2^ being minimized. All non-H atoms were refined with anisotropic displacement parameters, while H atoms were constrained to parent atom sites using a riding model (aromatic C–H = 0.95 Å; aliphatic C-H = 0.98 Å). The graphics were generated using the DIAMOND (version 3.0c, Crystal Impact, Bonn, Germany) Visual Crystal Structure Information software [35], with 50% displacement ellipsoids for non-hydrogen atoms.

### 3.6. Biological Study

#### 3.6.1. Cell Culture

The growth inhibitory effects of the title compound were evaluated using a panel of three cell lines: breast cancer cells (MCF-7), prostate cancer cells (PC-3), and non-cancerous fibroblasts (MRC-5), all obtained from the American Type Culture Collection (ATCC, Manassas, VA, USA) [36,37]. Cells were initially cultured in T-25 flasks (Thermo Scientific, Waltham, MA, USA) in Dulbecco’s Modified Eagle Medium (DMEM) supplemented with 10% fetal bovine serum (FBS) and penicillin–streptomycin–neomycin (PSN) [38,39], and maintained at 37 °C in a humidified incubator with 5% CO_2_ [40]. The culture medium was refreshed every two days, and once the cells reached approximately 80% confluence, they were seeded into 96-well plates for cytotoxicity assays [41]. Dichloromethane (DCM), the solvent used to dissolve the compound, was included as a negative control, while camptothecin served as a positive control.

#### 3.6.2. Cell Viability Assay

To evaluate the impact of the parent molecule on the aforementioned cancer cell lines, the seeded cells were treated with 1.53 µM, 3.125 µM, 6.25 µM, 12.5 µM, and 25 µM of the synthesized molecule in 1% Dichloromethane (DCM). DCM was used as a negative control, and the cells were incubated at 37 °C with 5% CO_2_ for 22 h. Following incubation, 10 µL of Alamar Blue was added to each well and incubated for an extra two hours.

A plate reader was then used to read the fluorescence at 530/25 excitation and 590/25 emission wavelengths, and the cell viability was calculated using the following equation:

$$\%\;\mathrm{Cell}\;\mathrm{viability}\;=\;\frac{Fluorescence\;of\;treated\;cells}{Fluorescence\;of\;untreated\;cells}\;\times \;100$$

A concentration-dependent response curve was plotted using GraphPad Prism v8.0 software to generate the IC_50_ concentrations [42,43].

#### 3.6.3. Statistical Analysis

For this study, all the experiments were performed in triplicate, and the results were analysed using GraphPad Prism8.4.2: one-way ANOVA, multiple comparisons wherein *p* < 0.05 was found to be statistically significant. The asterisks (*) signify the degree of significance, whereby (**) means moderately significant, (***) means highly significant, and (****) means very highly significant.

### 3.7. Molecular Docking Studies

The molecular docking was done using the Schrödinger Suite Release 2025-3 [44]. The target proteins were selected in a way that ensures they are representatives of the two cancer lines under examination. The PDB IDs, 5L2S, 5U1X, 6OAC, 4GV1, and 4JSV are used in the study. These protein complexes serve as representatives of the prostate and breast cancer lines used in this study [45]. The 5L2S is a PI3Kα (p110α) in complex with GDC-0941 (PI3K inhibitor). PI3K signaling is frequently dysregulated in ER+ breast cancer and contributes to proliferation/survival. The 5U1X represents the P2X4 receptor with bound antagonist. P2X purinergic receptors modulate inflammation and tumor microenvironment in solid tumors. The 6OAC is HDAC6 with ricolinostat-like inhibitor, where the HDAC6 is often overexpressed in breast cancer and AML, regulating epigenetic control and immune responses. The 4GV1 is the CDK4/cyclin D1 complex with an abemaciclib-like inhibitor. The CDK4/6 drives cell-cycle progression in ER+ breast cancer and is a validated target. The 4JSV represents the crystal structure of the mammalian target of rapamycin (mTOR) kinase, which is a critical regulator of cell growth, metabolism, and survival. These targets were chosen for their biological relevance to the studied cell lines and availability of high-resolution co-crystal structures with bound ligands suitable for docking validation.

For preparing the target proteins for the docking exercise, the Protein Preparation Wizard module in Maestro version 13.6 is used. The Wizard automates the complex process to ensure the protein structure is chemically and sterically correct for subsequent calculations. The PDB file of the protein is first loaded into Maestro, and the wizard automatically identifies issues such as missing atoms, incorrect bond orders, or unexpected bond angles. It then assigns the correct bond orders to the protein and adds all missing hydrogen atoms [46]. The OPLS4 force field was used to execute restricted energy reduction [47].

The LigPrep tool in Schrödinger (New York, NY, USA) was used to prepare the ligands. To be able to dock the ligands on the proteins, the binding site for each ligand on the protein of interest was created using Schrödinger’s Glide Receptor Grid Generation tool. The prepared ligands were docked on this grid using the Ligand Docking Wizard. All other settings were left alone, and the docking process was run in extra precision (XP) mode [48].

## 4. Conclusions

In this study, 2-((4-morpholino-1,2,5-thiadiazol-3-yl)oxy)benzaldehyde was successfully synthesized via a nucleophilic aromatic substitution reaction between salicylaldehyde and 3-chloro-4-morpholino-1,2,5-thiadiazole under basic conditions. The structure of the synthesized compound was confirmed by comprehensive spectroscopic and analytical techniques, including NMR, EA, and single-crystal X-ray diffraction, which collectively verified the formation of the desired aryl ether linkage. Preliminary biological study revealed that the compound exhibits promising bioactivity, suggesting that the thiadiazole–morpholine framework may serve as a valuable scaffold for the development of new pharmacologically active agents. Further structural optimization and mechanistic studies are warranted to enhance its potency and explore its therapeutic potential.

## Data Availability

The crystallographic data for this study have been deposited with the Cambridge Crystallographic Data Centre (CCDC) under deposition number 2495431. This data can be obtained free of charge from the CCDC via www.ccdc.cam.ac.uk (accessed on 25 October 2025).
